# Efficacy of LAMB against Emerging Azole- and Multidrug-Resistant *Candida parapsilosis* Isolates in the *Galleria mellonella* Model

**DOI:** 10.3390/jof6040377

**Published:** 2020-12-18

**Authors:** Ulrike Binder, Amir Arastehfar, Lisa Schnegg, Caroline Hörtnagl, Suleyha Hilmioğlu-Polat, David S. Perlin, Cornelia Lass-Flörl

**Affiliations:** 1 Institute of Hygiene and Medical Microbiology, Medical University Innsbruck, Schöpfstrasse 41, 6020 Innsbruck, Austria; lisa.schnegg@student.i-med.ac.at (L.S.); caroline.hoertnagl@i-med.ac.at (C.H.); cornelia.lass-floerl@i-med.ac.at (C.L.-F.); 2 Center for Discovery and Innovation, Hackensack Meridian Health, Nutley, NJ 07110, USA; a.arastehfar.nl@gmail.com (A.A.); David.Perlin@hmh-cdi.org (D.S.P.); 3 Division of Mycology, University of Ege, 35100 Izmir, Turkey; suleyha56@gmail.com

**Keywords:** azole-resistant, micafungin-resistant, *Candida parapsilosis*, liposomal amphotericin B, *Galleria mellonella* model

## Abstract

While being the third leading cause of candidemia worldwide, numerous studies have shown severe clonal outbreaks due to fluconazole-resistant (FLCR) *Candida parapsilosis* isolates associated with fluconazole therapeutic failure (FTF) with enhanced mortality. More recently, multidrug resistant (MDR) *C. parapsilosis* blood isolates have also been identified that are resistant to both azole and echinocandin drugs. Amphotericin B (AMB) resistance is rarely reported among *C. parapsilosis* isolates and proper management of bloodstream infections due to FLZR and MDR isolates requires prompt action at the time of outbreak. Therefore, using a well-established *Galleria mellonella* model, we assessed whether (a) laboratory-based findings on azole or echinocandin (micafungin) resistance in *C. parapsilosis* lead to therapeutic failure, (b) LAMB could serve as an efficient salvage treatment option, and (c) distinct mutations in *ERG11* impact mortality. Our in vivo data confirm fluconazole inefficacy against FLCR *C. parapsilosis* isolates carrying Y132F, Y132F + K143R, Y132F + G307A, and G307A + G458S in Erg11p, while LAMB proved to be an efficacious accessible option against both FLCR and MDR *C. parapsilosis* isolates. Moreover, positive correlation of in vitro and in vivo data further highlights the utility of *G. melonella* as a reliable model to investigate azole and polyene drug efficacy.

## 1. Introduction

Although *Candida parapsilosis* ranks as the third leading cause of candidemia worldwide [1], it is the second most prevalent *Candida* species causing candidemia in Kuwait [2], China [3], Japan [4], Spain [5], Greece [6,7], South Africa [8], and Latin American countries [9]. Moreover, this species has been reported as the leading cause of candidemia in some single center studies [10]. Contrary to the low levels of fluconazole resistance observed for *C. parapsilosis* blood isolates globally [1], recent studies from the USA [11], South Korea [12], Brazil [13], India [14], Italy [15], South Africa [16], and Turkey [17,18] have shown a high rate of clonal expansion of fluconazole-resistant (FLCR) *C. parapsilosis* isolates in clinical settings. Indeed, development of FLCR *C. parapsilosis* isolates occurs rapidly following antifungal treatment leading to fluconazole therapeutic failure, which necessitates switching to other systemic antifungals [19]. Surprisingly, new studies from Turkey [17,18] and South Korea [20] reported a substantial increase in prevalence of FLCR *C. parapsilosis* after 2015, which appeared partially due to the expanded use of azoles in clinical settings. Despite extremely low rates of echinocandin-resistant *C. parapsilosis* blood isolates [1], recent candidemia studies from Iran [21] and Turkey [22] have shown emerging multidrug-resistant (MDR) *C. parapsilosis* isolates resistant against both echinocandins and azoles. Of particular concern is the horizontal transfer of clonal FLCR and MDR *C. parapsilosis* to antifungal-naïve patients [18,22], which notoriously limit the efficacy of these two most widely used antifungals.

Fluconazole resistance primarily involves alteration of the drug target, *ERG11*, by specific point mutations, including Y132F, K143R, and G458S [23]. Other determinants, such as overexpression of efflux pumps and *ERG11* cooperatively contribute to azole resistance [23]. Resistance to echinocandins in *Candida* species are orchestrated by point mutations occurring in the hotspots (HS) of *FKS* genes, but until recently this phenomenon has not been reported for clinically obtained echinocandin-resistant isolates of *C. parapsilosis* [22].

New late-stage clinical trial antifungal drugs, like the echinocandin rezafungin, show promising in vitro and in vivo properties [24]. Therefore, identifying efficacious antifungals from the existing drug repertoire routinely used in the clinic will allow us to rapidly respond to this growing concern. Interestingly, despite identifying numerous FLCR and some MDR *C. parapsilosis* blood isolate during the last 13 years in Turkey [17,18,22], none of these isolates were resistant against amphotericin B (AMB) [18,22]. Additionally, the lipid formulation of AMB, LAMB, proved to be efficacious in eradicating invasive fungal infections even in patients suffering from acute kidney injury [25]. Further, AMB is well tolerated among neonates [26] as the most susceptible patient groups developing candidemia due to *C. parapsilosis* [27].

Determination of in vivo efficacy of antifungals against genetically defined fungal species can be achieved using a wide range of animal models. Although rodents present the most reliable models to evaluate the in vivo efficacy of antifungals, their use is limited by high costs, ethical constraints, and low throughput [28]. *Galleria melonella*, however, represents a suitable alternative [28]. Therefore, herein we explored the in vivo efficacy of fluconazole (FLC), micafungin (MFG), and LAMB against FLCR, and MDR *C. parapsilosis* isolates, harboring distinct mutations in *ERG11* and *FKS12*.

## 2. Materials and Methods

### 2.1. C. parapsilosis Isolates and Clinical Data

In order to explore the in vivo efficacy of FLC, MFG, and liposomal amphotericin B (LAMB), we used six *C. parapsilosis* blood isolates recovered during persistent outbreaks in Ege University Hospital, Izmir, Turkey, between 2007 and 2020, which had various antifungal susceptibility patterns (Table 1). The in vitro susceptibility profiles and amino acid (AA) substitutions in *ERG11* and *FKS1* in these strains, plus pertinent clinical data of patients infected with these isolates, are presented in Table 1. Except for patients infected with CP30 and CP37, those infected with CP70, CP179, CP207, and L48 have been reported previously [17,18,22]. A *C. parapsilosis* (ATCC 22019) isolate susceptible to all antifungals was used as a control strain in all assays. Therapeutic failure resulted when fever persisted and blood culture yielded *C. parapsilosis* despite antifungal therapy [29]. Antifungal susceptibility testing was carried out by the broth microdilution method suggested by CLSI M27-A3/S4 [30,31]. Details of microsatellite typing are reported in our previous studies [18,22].

### 2.2. Galleria mellonella In Vivo Treatment Studies

Sixth instar larvae of *Galleria mellonella* (SAGIP, Italy), weighing between 0.4 and 0.5 g, were infected with 10^7^ cells of the respective *C. parapsilosis* strain and received antifungal treatment 2 h post infection according to Maurer et al. [32] Larvae received a single dose of 5 μg (15 mg/kg) of each antifungal agent. To validate the *Galleria* model for predicting therapeutic outcome in *C. parapsilosis* infections, strain ATCC2209, which is susceptible to all antifungal agents and serves as the recommended control strain by the European Committee on Antimicrobial susceptibility testing (EUCAST) guidelines, was used to test concentration dependent efficacy of the respective drug (Supplementary Materials, Figure S1). Positive treatment outcome in FLC-treated larvae infected with the susceptible control strain, indicate that the concentration of FLC chosen was sufficient to cure the infection. Larvae infected with this strain also responded well to LAMB, which not only correlates well to in vitro antifungal susceptibility patterns, but also agrees with previous studies in which LAMB was shown to be efficient against *Aspergillus spp*. infections [33].

FLC concentrations were chosen based on previous work, in which 5 μg FLC were shown to be efficient against *C. orthopsilosis* [34], a close relative of *C. parapsilosis*; this concentration resembles the maximum dose for humans recommended by EUCAST rational version 3.0. As the recommended maximum human dose did not show treatment effect for LAMB and MFG (Figure S1B,C), the concentration was raised to 5 x of the most commonly used dosage recommended by EUCAST. None of the antifungal used showed toxic effects on larval survival at the concentrations used. Larvae were incubated at 37 °C and survival was monitored every 24 h over a period of 6 days (144 h). Untouched larvae and larvae injected with sterile phosphate buffered saline (PBS) served as controls; fungal cells and antifungals were diluted in sterile PBS. For each test group, 20 larvae were used (seven strains and three antifungals, namely FLC, MFG, and LAMB); experiments were repeated at least three times. Significance was determined with log-rank (Mantel–Cox) test, utilizing GraphPad Prism 7.00 software. Differences were considered significant at *p*-values ≤ 0.05.

## 3. Results

### 3.1. In Vitro Antifungal Susceptibility Pattern and Alterations in ERG11 or FKS1

Previous studies revealed a growing number of FLCR *C. parapsilosis* isolates in Turkish hospitals [17,18,22]. Therefore, we chose to investigate some of these clinical isolates that exhibited resistance to FLC and voriconazole (VRC), and harbored the most prevalent and/or novel mutations in *ERG11*. The majority of FLCR isolates showed previously described mutations in *ERG11*, such as Y132F or K143R substitutions or both (CP30, CP37, CP207). Interestingly, strain CP179 and strain L48 exhibited an additional, novel AA substitution, namely G307A, which has not been described previously and was a second AA exchange next to one already described (Y132F for CP179, and G458S for L48) [17,18,22]. As no isolates were found with G307A as the only AA substitution, and we did not perform genetic modification to prove the impact of this single exchange on FLC resistance, we do not know if this exchange of AA confers FLC resistance on its own. One isolate, CP70, exhibits resistance to FLC, but no other antifungal drug tested was wild-type (WT) for *ERG11*. The one MDR isolate chosen, CP207, exhibited an R658G AA substation in the Fks1 gene in addition to the already described Y132F + K143R changes in Erg11, and was therefore an MDR *C. parapsilosis* isolate (Table 1).

### 3.2. In Vivo Efficacy of Antifungal Treatment

**Figure 1:**
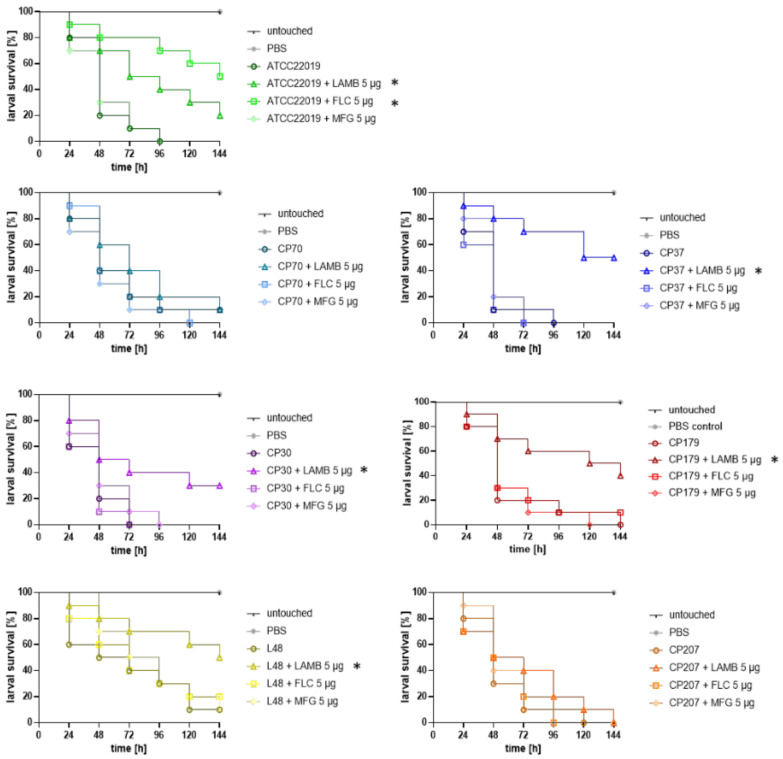
Ability of antifungal drugs to improve survival of *G. mellonella* larvae infected with *C. parapsilosis* isolates. All isolates except ATCC22019 showed in vitro resistance against FLC. CP30, CP179, L48 were additionally classified resistant against VRC. CP207 is a multidrug resistant (MDR) isolate with MFG resistance in addition to azole resistance. Larvae were infected with 10^7^ cells of the respective strain and treated groups received a single dose of antifungal (LAMB, FLC, or MFG) 2 h post infection (15 mg/kg). Control groups and untreated groups received the same volume of PBS (20 μL). * Indicates statistical difference (*p* < 0.05) according to log-rank (Mantel–Cox test) in comparison to the survival of larvae receiving no antifungal drug. LAMB: liposomal amphotericin B, FLC: fluconazole, MFG: micafungin, VRC: voriconazole.

To determine whether in vitro resistance correlated with in vivo FLC or MFG resistance, hence therapeutic failure, we took advantage of a *G. mellonella* model of invasive candidiasis. In vitro susceptibility patterns and in vivo outcome, reflecting fluconazole efficacy, correlated for all strains (Figure 1). More precisely, while 5 μg FLC—a concentration that reflects the maximum daily therapeutic dose in humans—significantly increased survival of larvae infected with the susceptible control strain ATCC 22019, no improvement by FLC treatment in survival of larvae inoculated with *FLCR isolates* was seen (Figure 1) (log-rank test, *p* > 0.05), independent of the kind of AA substitutions. One-hundred percent mortality was reached for untreated larvae infected with ATCC22019, CP70, CP30, CP37, and CP 207 at day 4 (96 h) post infection. FLC treatment resulted in 70% survival of larvae at day 4 for the susceptible ATCC22019 strain, and even 50% of larvae survived by the end of the experiment, whereas no increase in survival was seen in the groups infected with resistant isolates. Two strains, CP179 and L48, exhibited slightly delayed mortality, resulting in 100% deaths at day 5, or 10% survival by the end of the experiment, respectively. In these groups, the mortality was the same in the FLC-treated group (CP179), or only 10% higher for L48, which did not yield statistically significant difference (log-rank test, *p* > 0.05). Interestingly, treatment failure also occurred in larvae infected with CP70, which shows positive correlation to in vitro resistance, although there was no mutation detected in *ERG11*, indicating other mechanisms of drug resistance such as overexpression of drug efflux pumps, which were not investigated in this study [23]. Remarkably, none of the infected larval samples, independent of the in vitro susceptibility profile of the strains to MFG, exhibited an increase in survival rate when receiving MFG (5 μg) (Figure 1). Although no toxic effects were observed in the control groups receiving MFG only, our data raise doubts on whether *Galleria* larvae are the ideal model to study MFG efficacy in vivo and further studies are needed to evaluate the utility of larvae to test echinocandine efficacy.

As MFG showed to be not an alternative treatment option at least in our model, LAMB was further tested as a salvage treatment option for strains exhibiting FLC or MFG resistance. Indeed, in vitro profiles of the respective strains matched in vivo outcome, as for all strains increase in survival rates was observed after receiving 5 μg (15 mg/kg) of LAMB, the concentration resembling three times the dosage used in humans, post infection (Figure 1). Survival was significantly increased in all strains (*p* = 0.001 for CP37; *p* = 0.044 for CP30, *p* = 0.0767 for CP 179 and *p* = 0.042 for L48), except CP70 (*p* = 0.545) and CP207 (*p* = 0.359). Although survival rate did not significantly improve for larvae infected with CP70 and CP207, time to death was prolonged by even a single dose of LAMB. For example, LAMB treatment resulted in 40% survival versus only 20% at day 3 for CP 70, and could improve survival by 30% in CP207-infected larvae at day 3. Efficacy of antifungal treatment in the case of FLC and LAMB was shown to be concentration dependent, with 5 μg being the most efficient dose against ATCC 22019 infection, leading to a significantly (*p* = 0.027 for LAMB and *p* = 0.001 for FLC) positive outcome in comparison to lower (0.25 and 2.5 μg) concentrations (Figure S1). For four strains, ATCC 22019, CP 37, CP30, and CP179, also lower concentration of LAMB, 2.5 μg led to a statistical increase in survival (*p* = 0.046, 0.002, 0.018, or 0.017, respectively; data not shown).

Finally, recent studies have shown that *C. parapsilosis* isolates carrying Y132F in Erg11 are presumptively associated with a higher mortality rate [17,18]. Therefore, we compared the survival rates of larvae infected with clinical strains to the ATCC-control strain, and to each other, and could not detect statistically significant differences in survival rates, depending on the respective AA substitution (Figure S2).

## 4. Discussion

The emerging FLCR *C. parapsilosis* isolates causing severe clonal candidemia outbreaks in numerous countries [8,10,12,15,16,18] and the sporadic MDR isolates resistant against both azoles and micafungin [21,22] highlight the importance of establishing an efficacious alternative treatment at the time of outbreak. Beyond fluconazole therapeutic failure, recent studies have shown that *C. parapsilosis* isolates carrying Y132F in Erg11 are presumptively associated with a higher mortality rate [17,18]. Therefore, the current study evaluated (a) whether laboratory-based findings on azole (FLC) or echinocandin (MFG) resistance in *C. parapsilosis* imply therapeutic failure, (b) the efficacy of LAMB as a salvage treatment option using a *G. mellonella* model of invasive candidiasis, and (c) if isolates carrying Y132F are more virulent than WT and FLCR isolates carrying other *ERG11* mutations. Our study highlights good correlation of in vitro and in vivo data for FLC and LAMB and the efficacy of LAMB as an alternative antifungal treatment for outbreak scenarios due to FLCR and MDR *C. parapsilosis* isolates. The utility of the model to assess echinocandin, or specifically MFG, efficacy needs to be further evaluated. Lastly, our *Galleria* model suggests that *C. parapsilosis* isolates harboring Y132F are not necessarily more virulent, which calls for murine models to more closely assess this observation.

FLC treatment clearly correlated with in vitro susceptibility profile of strains tested, meaning that all larvae infected with FLCR isolates showed therapeutic failure when treated with FLC. This observation may suggest that amino acid substitution found in Erg11p, including G307A and G458S, could confer azole resistance. G458S has been previously identified in an FLCR *C. orthopsilosis* isolate and proven to confer azole resistance [34]. This mutation corresponds to the G464S substitution in *C. albicans*, which was also proven to result in FLC resistance [35]. Studies evaluating various *ERG11* mutations in *C. albicans* have found that substitution of Glycine 307 to Serine in tandem with other mutations can have a significant impact on azole resistance relative to a single mutation [35]. Therefore, we assume that G307A may have similar impact on azole resistance in FLCR *C. paraspilosis* isolates. Indeed, our recent studies suggest that FLCR *C. parapsilosis* harboring G307A in tandem with Y132F and/or G458S has been increasingly recovered during outbreaks [17,18]. Finally, the FLCR *C. parapsilosis* isolate with WT *ERG11* was shown to harbor mutation in Tac1 (A352V) and Upc2 (A793S) [18]. Therefore, the overexpression of efflux pumps and *ERG11* may be a potential reason behind the therapeutic failure to azole in absence of *ERG11* mutation.

Reliability of the *Galleria mellonella* model for testing azoles has previously been shown for *A. fumigatus*. Forastiero et al. demonstrated a clear correlation of the in vitro and in vivo outcome for azole-resistant strains, and pharmacodynamic analysis revealed the area under the curve (AUC)/MIC ratio determined in hemolymph, comparable to previous murine studies. These data indicate that larvae are also a reliable model to test other azole agents [36]. Altogether, these data suggest *G. mellonella* as a reliable model to assess azole therapeutic failure for FLCR *C. parapsilosis* isolates and that FLC therapeutic failure can occur in FLCR *C. parapsilosis* WT isolates or with mutations in *ERG11*.

Failure of MFG to cure infected larvae regardless of the MFG susceptibility profiles of the isolates may have multifactorial reasons, for example, a fast clearance of the drug from the hemolymph or immunomodulatory effects. The latter have been observed by Fuchs et al. [37] who showed that MFG increased the number of hemocytes, the phagocytic cells in *Galleria* hemolymph, which led to protection against *Staphylococcus aureus* infections in this study. This could explain the finding that higher MFG concentrations caused larval death (unpublished results), potentially due to other unknown factors involving larval immune systems and suggests larvae are not the ideal model to study MFG efficacy, which is a certain drawback of this model system. Caspofungin, on the other hand, was shown to be effective in prolonging life in larvae infected with *C. albicans* in a previous study [38]. Of note, these in vivo observations do not undermine the inefficacy of candins to treat FLCR, fluconazole-susceptible (FLCS), and MDR isolates, but the incapability of *G. melonella* to assess the in vivo efficacy of echinocandins. As such, experiments involving murine models are required to more precisely evaluate the potency of candins against FLCR, FLCS, and MDR *C. parapsilosis* isolates.

In the next step, we used LAMB as a potential salvage treatment option, which resulted in positive correlation of in vitro susceptibility and treatment efficacy in vivo. Previous studies showed that LAMB is well tolerated by the larval host [22,32,33,39]. Therefore, LAMB can be considered as an efficacious and available salvage treatment when facing outbreaks caused by FLCR and MDR *C. parapsilosis* isolates. A hypothesis driven by antifungal susceptibility testing data from Ege University Hospital, where LAMB was used as a salvage treatment for FLCR and MDR *C. parapsilosis* isolates, and the fact that so far no AMB resistant isolates were described [17,18,22].

Finally, our *Galleria* model showed that *C. parapsilosis* isolates carrying Y132F are not more virulent compared to other strains, which is unlike the observations made in real-life [17,18] and murine models involving a higher number of Y132F and control (both FLCR and FLCS) *C. parapsilosis* isolates are necessary to rule out whether or not Y132F isolates are more virulent.

One limitation of the current study is that the role of single AA substitutions for FLC or MFG resistance was not assessed by precise genetic engineering, such as introducing single base mutation in the susceptible control strain. Only this could shed light on which AA exchange is responsible for the resistant phenotype, especially in those strains that bear more than one mutation. In addition, in this study we did not investigate the role of efflux pumps that could be the reason for FLC resistance of the isolate CP70.

Taken together, our study supports the use of LAMB as an efficacious alternative antifungal drug available to potentially achieve successful clinical outcomes in scenarios involving FLCR and MDR *C. parapsilosis* isolates. Nevertheless, murine studies are needed to underline our findings in an alternative model system. Moreover, our results reconsolidate the utility of *G. mellonella* as a reliable, alternative model to investigate the significance of in vitro azole resistance and if such phenotypes can be associated with therapeutic failure in real-life. The lack of higher virulence of *C. parapsilosis* isolates carrying Y132F warrants future studies to precisely define this counterintuitive observation.

## Figures and Tables

**Table 1:** Clinical data and microbiological properties of six clinical strains included in this study.

*Strain*	*Resistance Phenotype*	*Age/Sex*	*Underlying Conditions*	*AP*	*AT*	*Outcome*	*MIC (μg/mL)*				*AA Exchanges in Hot Spot Regions*	
							FLC	VRC	MFG	AMB	Fks1-HS1	Erg11
*CP30*	FLCR, VRCR	16 Y/M	Aplastic anemia	VRC	VRC	Died	**>32**	**1**	0.5	0.25 ^#^	WT	Y132F + K143R
*CP37*	FLCR	27 Y/F	Cardiomypathy	FLC	FLC	Died	**>32**	0.125	1	0.5	WT	Y132F
*CP 179*	FLCR, VRCR	3 M/F	Bowel perforation	None	MFG	Died	**>32**	**2**	0.5	0.5	WT	Y132F + G307A
*L48*	FLCR, VRCR	14 Y/F	Epileptic seizure and malnutrition	None	AMB and FLC	Survived	**>64**	**4**	1	1	WT	G307A + G458S
*CP70*	FLCR	64 Y/M	Cardiovascular complications	None	CSP	Died	**>32**	0.125	1	0.5	WT	WT
*CP207*	MDR	2 Y/M	Pneumonia, empyema	None	FLC	Survived	**>32**	**0.5**	**>8**	0.5	R658G	Y132F + K143R

^#^ MIC reading was done at 48 h, as growth in the control was insufficient at 24 h; AP: antifungal prophylaxis; AT: antifungal therapy; FLC: fluconazole; VRC: voriconazole, MFG: micafungin; AMB: amphotericin B; MIC minimal inhibitory concentration; WT: wild-type; Y: year; M: months.

## Supplementary Materials

The following are available online at https://www.mdpi.com/2309-608X/6/4/377/s1, Figure S1: In vivo efficacy of (A) FLC, (B) LAMB and (C) MFG in larvae infected with the susceptible *C. parapsilosis* control strain ATCC22019. Figure S2: Kaplan–Meier survival curves of larvae injected with the respective clinical isolate (10^7^ cells per larva), or ATCC22019 for comparison.
