# Supplementary material for: Efficacy of LAMB against Emerging Azole- and Multidrug-Resistant Candida parapsilosis Isolates in the Galleria mellonella Model
Source: J Fungi (Basel). 2020 Dec 18;6(4):377. doi: 10.3390/jof6040377 (PMC7767002; doi:10.3390/jof6040377)
Supplement: Supplementary file 1 [file jof-06-00377-s001.pdf]

Figure S1

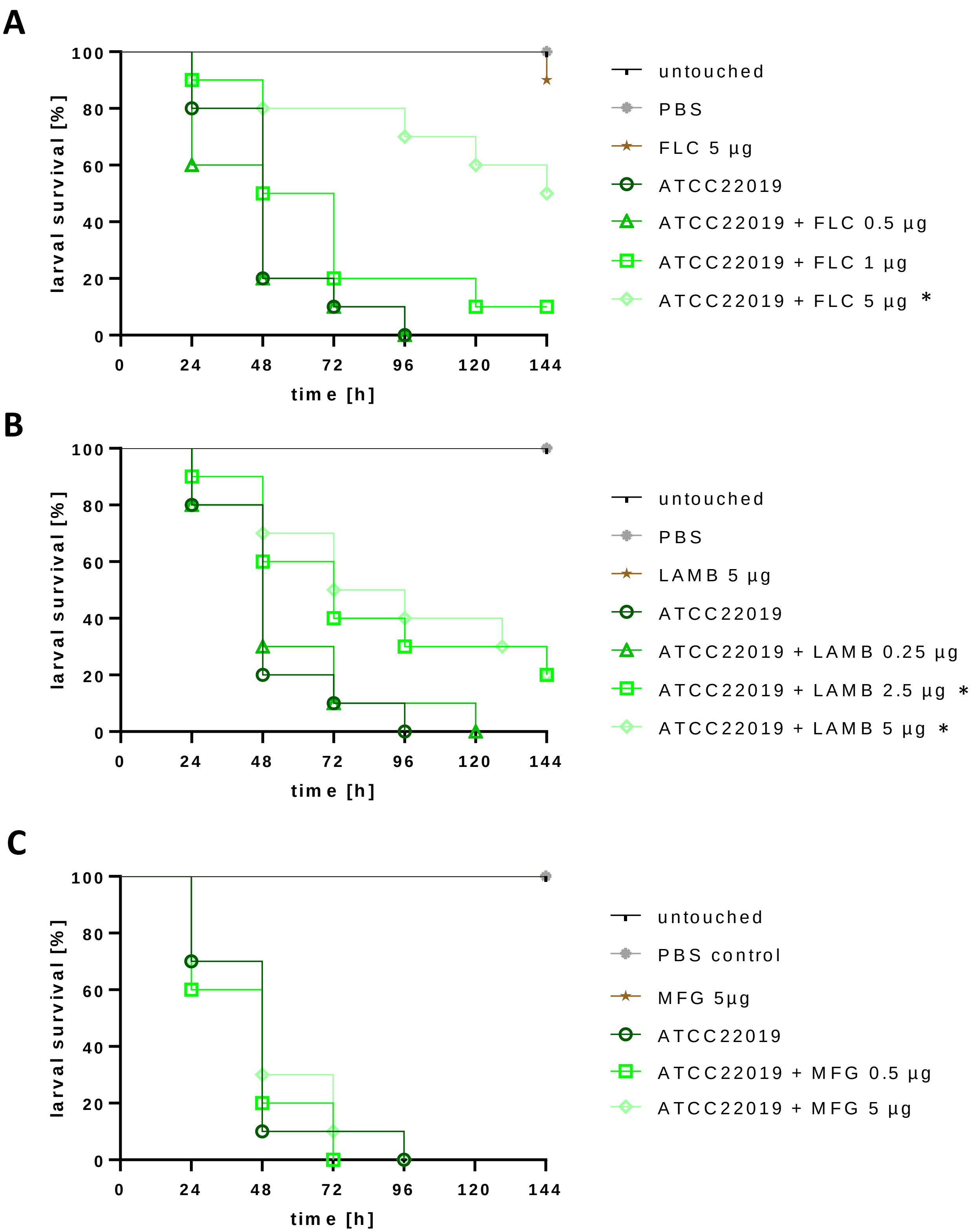

Figure S1: *In vivo* efficacy of **(A)** FLC, **(B)** LAMB and **(C)** MFG in larvae infected with the susceptible *C. parapsilosis* control strain ATCC22019. Infected larvae (10^7 cells per larva) received antifungal treatment 2h post infection. PBS was used as injection control and larvae receiving no treatment served as controls. Treatment with 5µg FLC, 2.5 µg LAMB and 5 µg LAMB resulted in significantly (p=0.001; 0.046 and 0.027, respectively) higher survival rates (indicated by *) compared to untreated control larvae. MFG treatment showed no curative effect on larvae.

Figure S2

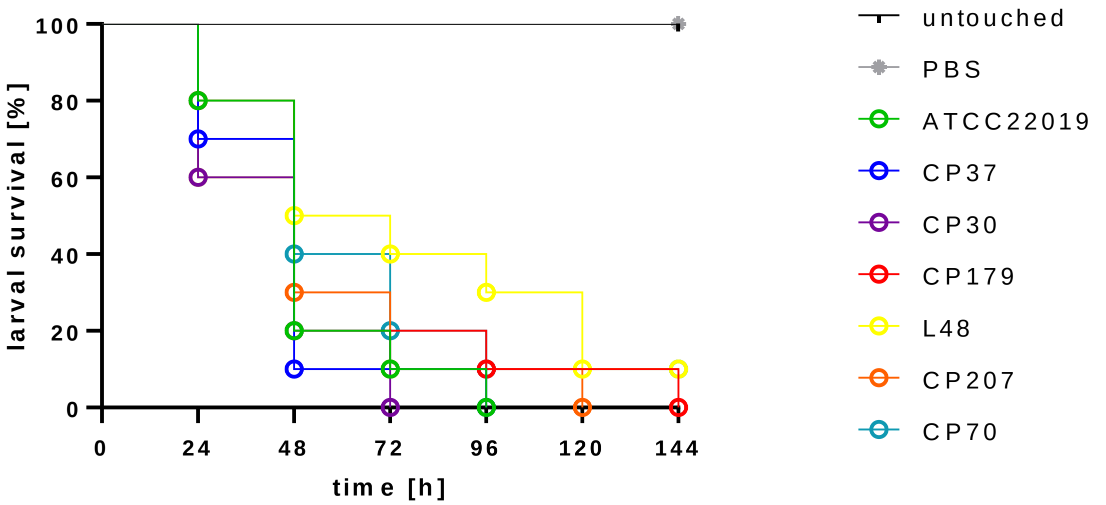

Figure S2: Kaplan Meyer Survival curves of larvae injected with the respective clinical isolate (10^7 cells per larva), or ATCC22019 for comparison. None of the strains exhibited statistically significant ($p \leq 0.05$) different mortality in comparison to ATCC22019 or to any of the other strains.
